# Beneficial Effect of High-Frequency Repetitive Transcranial Magnetic Stimulation for the Verbal Memory and Default Mode Network in Healthy Older Adults

**DOI:** 10.3389/fnagi.2022.845912

**Published:** 2022-05-04

**Authors:** Dong Cui, Jingna Jin, Weifang Cao, He Wang, Xin Wang, Ying Li, Tianjun Liu, Tao Yin, Zhipeng Liu

**Affiliations:** ^1^Institute of Biomedical Engineering, Chinese Academy of Medical Science and Peking Union Medical College, Tianjin, China; ^2^Department of Radiology, Shandong First Medical University and Shandong Academy of Medical Sciences, Tai’an, China; ^3^Neuroscience Center, Chinese Academy of Medical Science and Peking Union Medical College, Beijing, China

**Keywords:** repetitive transcranial magnetic stimulation, verbal memory, default mode network, aging, functional magnetic resonance imaging

## Abstract

Repetitive transcranial magnetic stimulation (rTMS) of the dorsolateral prefrontal cortex (DLPFC) is a non-invasive effective treatment for cognitive disorder, but its underlying mechanism of action remains unknown. The aim of this study was to explore the effect of a 2-week high-frequency (HF) active or sham 10 Hz rTMS on verbal memory in 40 healthy older adults. Resting-state functional magnetic resonance imaging (rs-fMRI) was used to measure functional connectivity (FC) within the default mode network (DMN). Verbal memory performance was evaluated using an auditory verbal learning test (AVLT). Additionally, we evaluated the relationship between memory improvement and FC changes within the DMN. The results revealed that HF-rTMS can enhance immediate recall and delayed recall of verbal memory and increased the FC of the bilateral precuneus (PCUN) within the DMN. The positive correlations between the immediate recall memory and the FC of the left PCUN after a 2-week intervention of HF-rTMS were detected. In conclusion, HF-rTMS may have the potential to improve verbal memory performance in older adults, which relation to FC changes in the DMN. The current findings are useful for increasing the understanding of the mechanisms of HF-rTMS, as well as guiding HF-rTMS treatment of cognitive disorders.

## Introduction

Aging is a major risk factor for many age-related diseases, such as cognitive impairment or dementia, and has become a growing public health problem. The progressive decline of cognitive functions (such as execution, attention, and memory) is a characteristic of normal brain aging (Singer et al., 2003; Deary et al., 2009; Stanziano et al., 2010; Toepper, 2017). Memory loss is the primary manifestation of early cognitive impairment in older adults (Jonker et al., 2000). Healthy older adults tend to be less efficient at encoding information and have more difficulty with delayed recall than young adults (Guo et al., 2007). Episodic memory is considered to be the form of long-term memory that displays the largest degree of age-related decline (Nyberg et al., 2003). A study of predictors of Alzheimer’s disease (AD) reported that episodic memory significantly decreased before the onset of clinical symptoms of dementia (Chen et al., 2001). Moreover, visuospatial and verbal memory performance is also reduced in older adults (Park et al., 2002). The vast majority of older adults suffer declines in cognitive functions, interfering with their ability to participate in meaningful activities and reduces their quality of life (Yu et al., 2021). A successful strategy to promote healthy brain aging is therefore of great interest to public health efforts and the economy.

Brain aging leads to cognitive decline, and this decline is also associated with a functional reorganization of the human brain. The default mode network (DMN) implicated in memory, and interactions between the DMN and frontoparietal executive control network (FPCN) were shown to support mnemonic processing (van Buuren et al., 2019). Task-based interactions between the DMN and FPCN were found to contribute to rapid memory retrieval and memory search (Kragel and Polyn, 2015). Previous studies on human brain aging have shown that functional connectivity (FC) within higher-order resting-state brain networks (i.e., DMN) decreases while, between-network connectivity (i.e., DMN and FPCN) increases in older age (Song et al., 2014; Geerligs et al., 2015; Iordan et al., 2017; Schlesinger et al., 2017). In addition, these changes have been associated with less efficient cognitive functioning. DMN activity showed a significant negative correlation with age and was associated with decreased attention, memory, and executive functions (Damoiseaux et al., 2008). And a longitudinal study showed an increase in functional integration between DMN and FPCN, which was also related to lower processing speed (Ng et al., 2016). These results highlight the critical importance of this organizational characteristic of the aging brain.

In the past decade, transcranial magnetic stimulation (TMS) has been used in neuroscience to investigate the intervention of human cognitive function (Kobayashi and Pascual-Leone, 2003; Rossini and Rossi, 2007; Eldaief et al., 2013). An increasing number of studies have shown that repetitive TMS (rTMS) can modulate memory function and brain activities. A meta-analysis of the older adults and those with clinical disorders revealed that 5 and 10 Hz offline rTMS protocols had enhancing effects onepisodic memory (Yeh and Rose, 2019). Recent studies showed that high-frequency rTMS (HF-rTMS) applied over the left dorsolateral prefrontal cortex (DLPFC)can enhance working memory performance in healthy older adults (Beynel et al., 2019a, b; Lefaucheur et al., 2020). Voss and colleagues found that HF-rTMS on the left parietal lobe improved associative memory performance in healthy young adults (Wang et al., 2014; Wang and Voss, 2015). Moreover, the improvement of associative memory was significantly correlated with FC changes of the hippocampus with precuneus cortex (PCUN), fusiform gyrus (FG)/parahippocampal (ParaHIP) cortex, parietal cortex, and left lateral parietal cortex. Similarly, Nilakantan also found a significant correlation between age-related recall memory impairments and hippocampal-cortical FC in older adults (Nilakantan et al., 2019). These results suggest a causal relationship between human brain networks and specific memory functions. Previous studies have found that rTMS over the DLPFC has a significant regulatory effect on brain regions within the DMN. Shang et al. (2020) found that the rTMS reduces FC between the continuous theta burst stimulation (cTBS) target: the left DLPFC and brain regions within the DMN. In another study, they found that HF-rTMS can increase relative cerebral blood flow (rCBF) in the left medial temporal lobe/hippocampus in the DMN (Shang et al., 2018). HF-rTMS significantly increased the amplitude of low-frequency fluctuation (ALFF) in the anterior cingulate cortex (ACC) and the medial prefrontal cortex (mPFC) and significantly improved the FC of the ACC with the right medial superior frontal gyrus (SFG), inferior temporal gyrus (ITG), and left superior temporal gyrus (STG), and angular gyrus (AG) were also observed (Xue et al., 2017). However, the neural mechanism of rTMS remains unclear. To date, no studies have explored whether the regulation of rTMS on memory function in older adults is related to the change in brain network activity.

Previous studies have shown that DLPFC is a common target for non-invasive brain stimulation (NIBS) in healthy and clinical research (Brunoni and Vanderhasselt, 2014; Martin et al., 2017) because it is a critical region in cognitive control. And cognitive control, a key aspect of executive functioning, is closely related to mental health and consists of a series of multiple cognitive stages of perceptual processing, attention capture, conflict monitoring, and conflict interference resolution among others (Blumenfeld and Ranganath, 2006; Millner et al., 2012). Therefore, DLPFC was selected as the target of HF-rTMS in this study. Healthy older adults (65–75 years of age) were recruited to investigate the FC changes within the DMN before and after HF-rTMS and their relationship with the improvement of verbal memory and to gain insights into the neural mechanisms of verbal memory decline in older adults during aging using rs-fMRI. The study of brain network alterations in verbal memory improvement with rTMS provides the theoretical foundation for the clinical treatment of AD and other neurodegenerative diseases with rTMS.

## Materials and Methods

### Participants

A total of 52 older adults (65–75 years of age) were recruited from the local community from August 2019 to December 2019. All of the subjects were right-handed, Han Chinese, and native speakers of Chinese, with ≥8 years of education.

Subjects were included if they had: (1) no mild cognitive impairment (MCI) or dementia according to the Diagnostic and Statistical Manual of Mental Disorders, 4th edition, revised (DSM-IV-R), and the Chinese Guidelines for Diagnosis and Management of Cognitive Impairment and Dementia (2011); (2) scored ≥26 on the Montreal Cognitive Assessment Basic (MoCA-B); (3) had a Clinical Dementia Rating (CDR) score = 0; (4) the assessment of activities of daily living (ADL) score = 100 (Mlinac and Feng, 2016); (5) no history of epilepsy or family history; and (6) no history of drug or alcohol abuse.

The exclusion criteria were as follows: (1) Body mass index ≥30 (Beyer et al., 2017); (2) Diabetes (Cui et al., 2019); (3) had a history of neurological or psychiatric disorders or traumatic brain injury or severe cardiovascular disease; (4) focal brain lesions on MRI images; (5) previous rTMS treatment; and (6) not met the safety standard of MRI scan and rTMS intervention.

**Figure 1:**
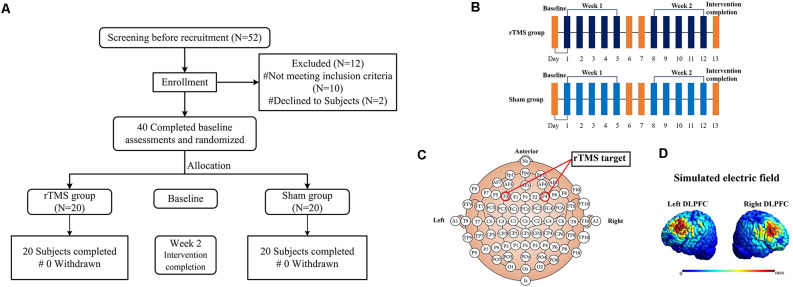
The flowchart **(A)**, diagrammatic representation of the timeline **(B)**, rTMS target **(C)** and simulated electric field modeling for rTMS over the left and right DLPFC **(D)** for this study. Note: all images in figure **(D)** show the electric field strength scaled from 0 (blue) to the individual maximum (red).

In all, two subjects were excluded for hearing impairment, one for alcohol abuse, four for MoCA-B score <26, two for overweight, two for diabetes, and one had a history of cardiac surgery. The 40 healthy older adults were age- and gender-matched and were randomly assigned to the real stimulation group (rTMS group, *n* = 20) and the sham stimulation group as a control (sham group, *n* = 20; as shown in Figure 1A). According to the Declaration of Helsinki, written informed consent was provided by all subjects or their representatives. This study was approved by the local ethics committees of the Institute of Biomedical Engineering, the Chinese Academy of Medical Sciences, and Peking Union Medical College.

### Neuropsychological Examination

All subjects in the present study were evaluated with a battery of neuropsychological tests at baseline and repeated at the end of the intervention (within 24 h). These tests were used to assess general cognitive ability and verbal memory. The Montreal Cognitive Assessment (MoCA) had been used to assess the level of general cognitive functioning, and we used the version MOCA-B in this study, for subjects with low education (Cao et al., 2012). Clinical dementia rating (CDR) and Self-rating depression (SDS) were used assess to the presence of dementia and depression (Jokelainen et al., 2019; Yang et al., 2021). Verbal Memory performance was further evaluated using an auditory verbal learning test (AVLT) for immediate recall, short-term delayed recall (about 5 min) and long-term delayed recall (about 20 min) verbal memory adapted to the Chinese population (Guo et al., 2007). Subjects memorize a semantically categorized list of 12 concrete nouns (consisting of two Chinese characters) that are read aloud by the neurologist at a rate of one word every 1.5 s. There were three semantic categories and four words in each category. Subsequently, subjects are asked to recall aloud as many words from the list as they can in any order. The immediate recall score (AVLT-1) is the sum of the correct words recalled by the subjects after three consecutive tests; after approximately 5 min and 20 min, participants are again asked to recall aloud as many words as possible from the previous list in any order. Short-term delayed recall memory (AVLT-2) was the number of correct words recalled after 5 min; Long-term delayed recall memory (AVLT-3) was the number of correct words recalled after 20 min. To prevent the learning effect, different words were used on the AVLT for the pre and post TMS sessions in this study.

### Stimuli and Procedure

A Magstim Rapid^2^ transcranial magnetic stimulator (Magstim, Whitland, UK) with a 70-mm air-cooled figure-of-eight coil was used to deliver sessions of rTMS. The intervention cycle in this study was five sessions per week for a total number of 2 weeks (10 sessions; as shown in Figure 1B). In all subjects, rTMS was applied over the left and right DLPFC. For localization of the DLPFC, the tip of the intersection of the two coil loops was lined up with the F3/F4 sites of the 10–20 EEG system (as shown in Figure 1C). According to a previous study, the Talairach coordinates of the stimulated cortical site corresponded approximately at ±40, 45, 28 (Turriziani et al., 2010). To accurately target the coil placement, all stimulations were guided by the subject’s anatomical 3D-T1images (1^*^1^*^1 mm^3^) and Brainsight neuro-navigation system (Rogue Research, Montreal, Canada). The coil and scalp into tangent placement and the direction of the coil handle was paralleled to the median sagittal plane. Simulated electric field modeling for rTMS over the left and right DLPFC was shown in Figure 1D (Thielscher et al., 2015). The rTMS parameters were as follows: Forty 10 Hz trains of 5 s at 110% of the resting motor threshold (RMT), which is in line with the international safety limits for use of rTMS (Rossi et al., 2009; Lefaucheur et al., 2020). RMT was defined as the lowest TMS intensity (as assessed by single-pulse TMS) able to induce a visible muscle twitch of the contralateral hand in at least 5/10 consecutive trials. RMT was determined on the same hemisphere of the stimulated DLPFC. There were no interhemispheric and group differences in RMT values (Table 1). Each DLPFC received 2,000-rTMS pulses per time of intervention for 20 min, changed the sequence of stimulation site every day and the intervention each time lasted for 40 min. For the sham group, the setting of parameters was the same as the rTMS group. And using a specially designed sham figure-of-eight coil (Magstim placebo coil system model, Magstim, Whitland, UK), which created identical noise as real rTMS but delivered no electromagnetic energy on the cortex. In order to protect the hearing of subjects from the noise generated by the transcranial magnetic stimulator, special earplugs were used during each session of rTMS. After each session, the subjects were asked about adverse reactions such as headache, toothache, facial twitching, tinnitus, palpitation, chest tightness, vertigo, or depression. All of the adverse events were recorded and are shown in Table 1.

**Table 1:** Sample characteristics.

Characteristics	rTMS group (*n* = 20)	Sham group (*n* = 20)	χ^2/T^	*p*
Age (years)	68.35 ± 3.31	67.95 ± 2.96	0.690^#^	0.420
Gender (male/female)	8/12	7/13	0.053^&^	0.818
Education (years)	10.55 ± 2.61	9.95 ± 2.24	0.782^#^	0.439
MoCA-B	27.85 ± 1.23	27.40 ± 0.95	1.303^#^	0.203
BMI	23.87 ± 2.60	24.92 ± 2.51	−1.296^#^	0.201
TC (mmol/l)	4.59 ± 0.54	4.54 ± 0.65	0.293^#^	0.771
FBG (mmol/l)	5.36 ± 0.63	5.51 ± 0.67	−0.738^#^	0.465
TIV (cm^3^)	1431.00 ± 159.35	1395.23 ± 107.51	0.832^#^	0.411
Resting motor threshold (RMT)				
Left hemisphere	54.90 ± 6.00	56.05 ± 5.86	−0.613^#^	0.543
Right hemisphere	53.55 ± 5.57	54.85 ± 5.74	−0.727^#^	0.472
Adverse reactions of rTMS (total times/number of subjects)				
Headache	4/3	1/1		
Toothache	1/1	1/1		
Facial twitching	1/1	-		
Tinnitus	-	1/1		

Note: Data expressed as mean ± SD; ^&^Chi-square analysis, ^#^Independent sample *T* test; ^*^p < 0.05. Abbreviations: BMI, body mass index; TC, total cholesterol; FBG, fasting blood glucose; TIV, total intracranial volume.

### MRI Data Acquisition

MRI data were collected on matched 3T Trio MR scanners (Siemens, Erlangen, German) using the 12-channel phased-array head coil at baseline and repeated at the end of intervention (within 24 h). Foam padding was used to minimize head motion for all subjects. Structural MRI data were obtained using a sagittal magnetization-prepared rapid gradient echo (MPRAGE) three-dimensional T1-weighted sequence with the following imaging parameters: repetition time *(TR)* = 6.1 ms, echo time *(TE)* = 2.0 ms, slice thickness = 1 mm, flip-angle = 9°, field of view (FOV) = 256 × 256 mm^2^, acquisition matrix = 256 × 256, slices = 176. An echo-planar imaging (EPI) sequence was applied to acquire the rs-fMRI data, the parameters were as follows: *TR* = 2,000 ms, *TE* = 30 ms, slice number = 30, slice thickness = 4 mm, flip-angle = 90°, matrix = 64 × 64, and FOV = 240 × 240 mm^2^, in-plane resolution = 3.75 mm × 3.75 mm. For each subject, a total of 250 volumes were acquired, resulting in a total scan time of 500 s. Subjects were instructed to keep their eyes closed, relax, not to think of anything in specific and not to fall asleep.

### Preprocessing of fMRI Data

SPM12^1^ and DPARSF 5.0^2^ were used for fMRI data pre-processing and batch processing. The steps included discarding the first 10 volumes to ensure steady-state longitudinal magnetization, slice timing, correction for head motion movement (subjects with translational or rotational motion higher than 2 mm or 2° were excluded), normalized to Montreal Neurologic Institute (MNI) space with voxel size of 3 × 3 × 3 mm^3^. Next, Friston 24-parameters head motion, white matter (WM) signals, and cerebrospinal fluid (CSF) signals were regressed out as nuisance covariates. Frame-wise displacement (FD) was calculated for each timepoint, subjects were excluded for further analyses if the mean *FD* > 0.5 mm (Power et al., 2012). Finally, spatially smoothed by convolution with an isotropic Gaussian kernel (FWHM = 6 mm).

### Independent Component Analysis

Group spatial ICA was conducted using the GIFT software^3^, version 4.0b). Dimension estimation was performed on all subjects using the minimum description length (MDL) criterion to determine the number of independent components (ICs). Then, fMRI data from all subjects were concatenated and the temporal dimension of the aggregate data set was reduced by means of principal component analysis (PCA). Subsequently, 38 ICs were identified in the rTMS group and sham group both at baseline and after intervention using the infomax algorithm. The ICASSO algorithm was used for stability analysis. To display the voxels that most strongly contributed to a particular IC, the intensity values in each spatial map were transformed into *Z* values. According to previous studies, it is normally accepted that *Z* values can indirectly provide a measure of FC within the network (Beckmann et al., 2005; Liao et al., 2010). Finally, the DMN, left FPCN, right FPCN, and salience network (SN) were selected based on the largest spatial correlation with reference templates (Mantini et al., 2007) and subjective judgment (Raichle et al., 2001; Raichle and Snyder, 2007; \hyperref[s9]**Supplementary Figure 1**). In addition, functional network connectivity (FNC) analysis was performed to calculate the functional connectivity between brain functional networks.

### Statistical Analysis

Demographic and clinical data were analyzed using SPSS 25.0 (IBM, USA). The Chi-square test (χ^2^) was used for gender. Two-sample *t*-tests were used for the continuous variables (age, years of education, and clinical data). Differences were considered significant at *P* < 0.05. We calculated the AVLT test score after TMS intervention minus that of the baseline, and then the general linear model (GLM) was used to analyze AVLT change differences between the rTMS and sham group, age, gender, and years of education were used as covariates. In addition, we compared the AVLT test score differences between the four groups. The IC corresponding to the DMN was extracted from all individuals of the four groups. The voxels belonging to the DMN in each group were identified by using one-sample *t*-test [*P* < 0.05, false discovery rate (FDR) corrected]. Spatial maps of the four groups were then combined to create a mask and were prepared for further analysis of variance (ANOVA; Zong et al., 2019). Repeated measure two factors ANOVA was performed to examine four group differences within the DMN, and gender, age, education level, and mean FD were included as covariates. Two factors included group factor (rTMS and sham) and time factor (baseline and weeks 2). We set the statistical significance level at a corrected *p*-value of 0.05 (FDR corrected) using the DPABI for the interaction effect and the two main effects. Group comparisons were limited to the voxels within the DMN mask, which were obtained from the results of one-sample *t-tests*. Several regions that exhibited significant abnormal FC of DMNwere selected as regions of interest (ROI) for *post-hoc* analyses (*P* < 0.05, FDR-corrected). Finally, age, gender, and years of education were used as covariates for Spearman correlation analysis between FC changes of DMN and verbal memory test scores with significant correlations (*P* < 0.05, FDR corrected).

## Results

### Sample Characteristics

As shown in Table 1, there were no significant differences between the rTMS and sham groups in age, gender, years of education, MOCA-B score, body mass index (BMI), total cholesterol (TC), hemoglobin A1C (HbA1c), fasting blood glucose (FBG), and total intracranial volume (TIV; *P* > 0.05). Adverse events during the intervention were also recorded in Table 1. Although adverse events did not affect the conduct of the rTMS intervention.

### AVLT Test Scores

**Figure 2:**
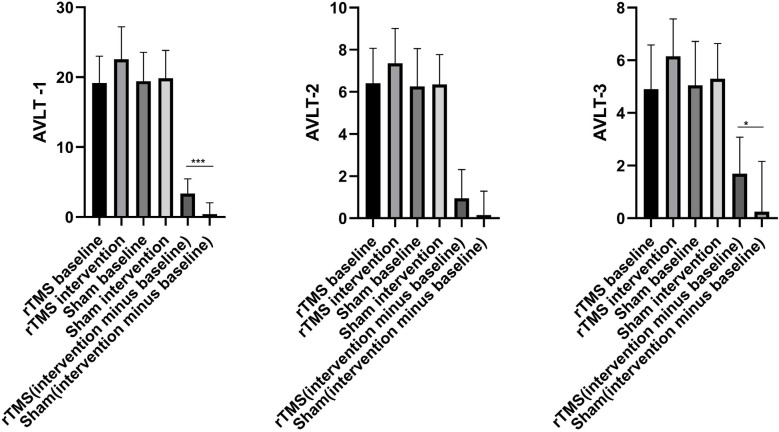
The histogram of group differences in AVLT between rTMS and Sham group. Note: Data expressed as mean ± SD; *p < 0.05, **p < 0.01, ^***^p < 0.001. Abbreviations: AVLT, auditory verbal learning test; AVLT-1, Immediate recall; AVLT-2, Delayed recall (5 min); AVLT-3, Delayed recall (20 min).

There were no significant differences in AVLT-1, AVLT-2, and AVLT-3 scores between the rTMS and sham groups at baseline (*P* > 0.05), as shown in \hyperref[s9]**Supplementary Table 1**. Two weeks after rTMS intervention, the AVLT-1, AVLT-2, and AVLT-3 scores were significantly different before and after intervention among subjects in the rTMS group, as shown in \hyperref[s9]**Supplementary Table 1**. Most notably, AVLT-1 and AVLT-3 scores differed significantly between the rTMS and sham groups (*P* < 0.05), as shown in Table 2; Figure 2.

**Table 2:** Group differences in AVLT between rTMS and Sham group.

Characteristics	rTMS baseline	rTMS intervention	Sham baseline	Sham intervention	rTMS (intervention minus baseline)	Sham (intervention minus baseline)	*F* ^#^	*p*	Cohen’s d	Effect size (r)
AVLT-1	19.20 ± 3.82	22.55 ± 4.65	19.45 ± 4.11	19.85 ± 3.99	3.35 ± 2.11	0.40 ± 1.64	21.58	#x0003C;0.001^***^	1.561	0.615
AVLT-2	6.40 ± 1.67	7.35 ± 1.66	6.25 ± 1.80	6.35 ± 1.42	0.95 ± 1.36	0.15 ± 1.14	3.931	0.055	0.638	0.304
AVLT-3	4.90 ± 1.68	6.15 ± 1.42	5.05 ± 1.67	5.30 ± 1.34	1.70 ± 1.38	0.25 ± 1.91	7.481	0.010^*^	0.870	0.399

Note: Data expressed as mean ± SD; ^*^p < 0.05, ^**^p < 0.01, ^***^p < 0.001. ^#^*F*, general linear model (GLM) was used to analyze AVLT change differences between the rTMS (intervention minus baseline) and s Sham (intervention minus baseline), age, gender, and years of education were used as covariates. Abbreviations: AVLT, auditory verbal learning test; AVLT-1, Immediate recall; AVLT-2, Delayed recall (5 min); AVLT-3, Delayed recall (20 min).

### Changes in Brain Functional Network and Correlation Analysis

**Figure 3:**
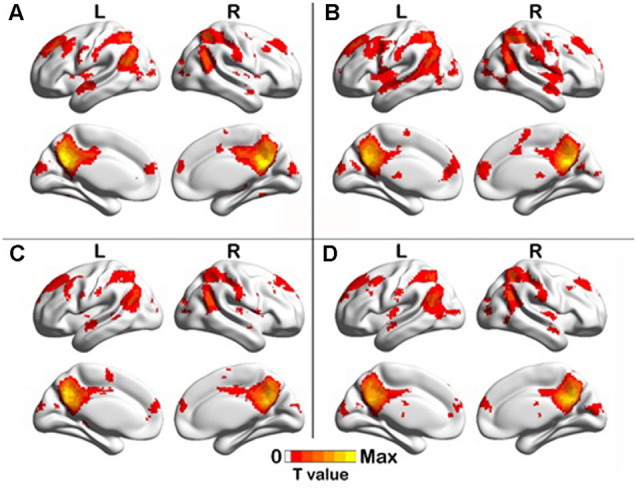
One-sample *T* test results of DMN. **(A)** rTMS baseline group, **(B)** rTMS intervention group, **(C)** Sham baseline group, **(D)** Sham intervention group (*p* < 0.05, FDR correction, voxels > 486 mm^3^).

**Figure 4:**
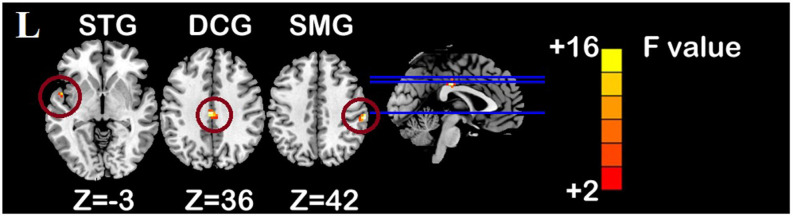
Interaction effect of functional connectivity changes within the DMN (*p* < 0.05, FDR correction, voxels > 486 mm^3^). Abbreviations: STG, superior temporal gyrus; SMG, supramarginal gyrus; DCG, median cingulate and paracingulate gyri; L, left hemisphere.

**Figure 5:**
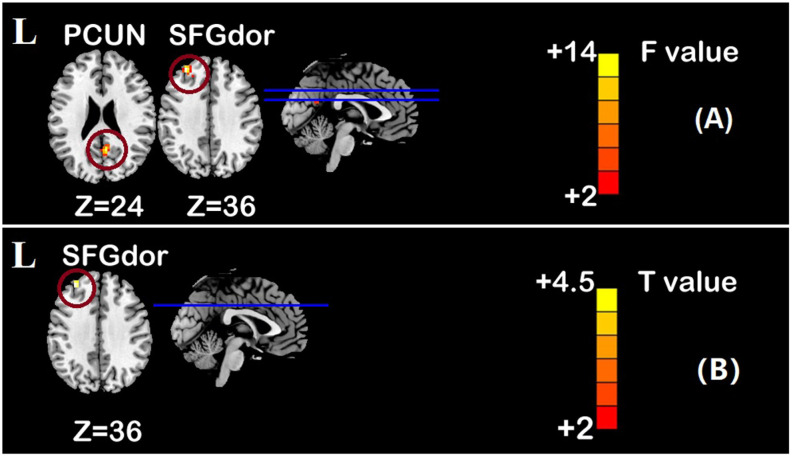
Group main effect of functional connectivity changes within the DMN and *post-hoc* analyses (*p* < 0.05, FDR correction, voxels > 486 mm^3^). **(A)** Group main effect, **(B)** rTMS intervention group vs. Sham intervention group. Abbreviations: PCUN, precuneus; SFGdor, dorsolateral superior frontal gyrus; L, left hemisphere.

**Figure 6:**
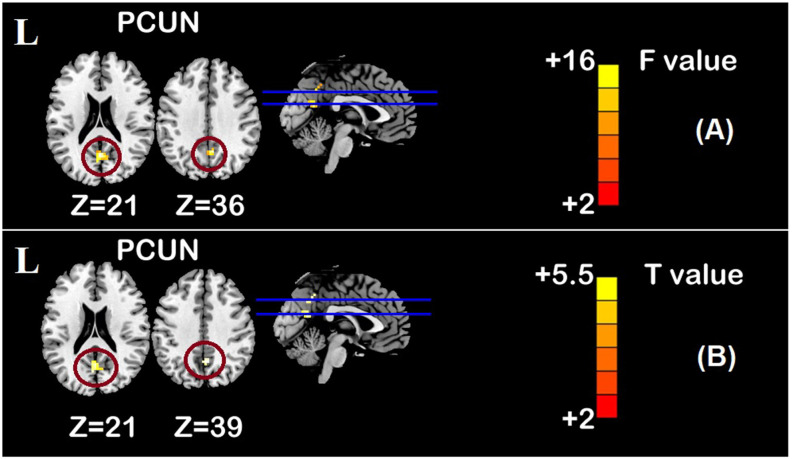
Time main effect of functional connectivity changes within the DMN and *post-hoc* analyses (*p* < 0.05, FDR correction, voxels > 486 mm^3^). **(A)** Time main effect, **(B)** rTMS intervention group vs. rTMS baseline group. Abbreviations: PCUN, precuneus; L, left hemisphere.

**Figure 7:**
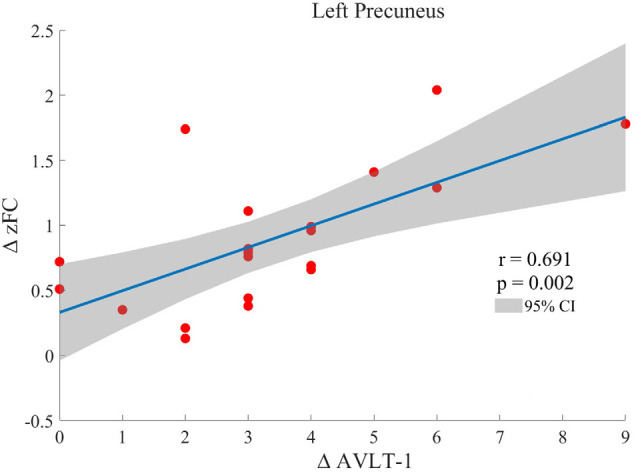
Spearman correlation between ΔzFC value of the left precuneus (after intervention minus baseline) and ΔAVLT-1 scores (after intervention minus baseline) in the rTMS group.

One-sample *t*-test (*P* < 0.05, FDR corrected, voxel >486 mm^3^) was used to observe the DMN of the two groups of older adults. At baseline and after rTMS intervention, the DMN mainly included the PCC, ventromedial prefrontal cortex (vPFC), DLPFC, PCUN, STG/middle temporal cortex (MTG), parietal cortex/angular gyrus (AG), and caudate nucleus (Figure 3; Gusnard et al., 2001; Raichle et al., 2001). In addition, we obtain the results of one-sample *t*-test of the left FPCN, right FPCN, and SN (\hyperref[s9]**Supplementary Figures 2–4**). Unexpectedly, significant interaction effects were observed only in the DMN. For significant FC changes within the DMN, group (rTMS and sham) by time (baseline and weeks 2) interaction effects were observed in the left medial paracingulate gyrus, left STG, and right supramarginal gyrus (SMG; Figure 4, Table 3; *P* < 0.05, FDR corrected, voxel >486 mm^3^). Furthermore, significant group main effects were observed in the right PCUN and left dorsolateral superior frontal gyrus (SFGdor; Figure 5A, Table 4), and time main effects were observed in the bilateral PCUN (Figure 6A, Table 5). In *post-hoc* analyses, FC of the left SFGdor within the DMN in the rTMS group was significantly higher than that in the sham group after intervention (Figure 5B; Table 4). Simultaneously, compared with baseline, FC of the bilateral PCUN increased significantly after intervention in the rTMS group (Figure 6B; Table 5). Spearman’s correlation revealed that ΔzFC value of the left PCUN (after intervention minus baseline) was significantly positively correlated with the ΔAVLT-1 score (after intervention minus baseline) in the rTMS group (*r* = 0.691, *P* = 0.002; Figure 7). Furthermore, there were no significant changes observed in the FNC analysis.

**Table 3:** Brain regions showing significant interaction effect of functional connectivity within DMN.

	Hemisphere	Regions	MNI			F/T	Voxels
			*X*	*Y*	*Z*		
Interaction effect	R	SMG	60	−24	42	15.83	28
	L	STG	−51	3	−3	13.70	31
		DCG	−3	−21	36	15.69	38

*Abbreviations: STG, superior temporal gyrus; SMG, supramarginal gyrus; DCG, median cingulate and paracingulate gyri; MNI, Montreal Neurological Institute*.

**Table 4:** Brain regions showing significant group main effect of functional connectivity within DMN and *post-hoc* analyses.

	Hemisphere	Regions	MNI			F/T	Voxels
			*X*	*Y*	*Z*		
Group effect*	L	SFGdor	−26	40	36	13.91	44
	R	PCUN	9	−57	24	13.44	39
rTMSintervention group>Shamintervention group^#^	L	SFGdor	−27	45	36	4.472	20

Note: *F value, ^#^*T* value. Abbreviations: PCUN, precuneus; SFGdor, dorsolateral superior frontal gyrus; MNI, Montreal Neurological Institute.

**Table 5:** Brain regions showing significant time main effect of functional connectivity within DMN and *post-hoc* analyses.

	Hemisphere	Regions	MNI			F/T	Voxels
			*X*	*Y*	*Z*		
Time effect*	R	PCUN	6	−57	36	15.84	37
	L	PCUN	0	−63	21	15.62	35
rTMS intervention group>rTMS baseline group^#^	R	PCUN	3	−57	39	5.299	27
	L	PCUN	0	−60	21	5.020	30

Note: *F value, ^#^*T* value. Abbreviations: PCUN, precuneus; MNI, Montreal Neurological Institute.

## Discussion

In this study, we used rs-fMRI and verbal memory tests (AVLT) to investigate the intervention effects of HF-rTMS on brain network and verbal memory in healthy older adults. We found that HF-rTMS could improve the verbal memory of healthy older adults and modulate the FC within the DMN. The increase in the FC of the left PCUN within the DMN was significantly positively correlated with immediate recall function, which suggests that the improvement of verbal memory by HF-rTMS may be related to the modulation of DMN activity.

Verbal memory decline is one of the major manifestations of aging and a key feature of AD. The AVLT is very sensitive in the detection of verbal memory impairment caused by various neurological and psychiatric diseases. For the recognition of MCI, the sensitivity of the AVLT is higher than that of the Rey Complex Figure Test (Zhao et al., 2015). The AVLT can also be used to evaluate neuroplasticity in older adults (Navarro and Calero, 2018). Our study showed that the immediate and delayed recall function of older adults was significantly improved after rTMS intervention, suggesting that HF-rTMS can effectively ameliorate the decline of verbal memory during aging. Similar results were observed in patients with MCI and AD before and after rTMS treatment, which further confirms the modulated effect of rTMS on verbal memory (Sabbagh et al., 2020; Mimura et al., 2021).

The DMN is one of the most studied brain networks in brain aging and neurodegenerative diseases (Beason-Held et al., 2017). Studies on early AD have focused on sensitive and specific biomarkers, which can help clinicians monitor the progress and treatment of AD. As a non-invasive neuroimaging biomarker for the diagnosis of early AD, DMN functional activity change has attracted increasing attention. Numerous neuroimaging studies showed that the activity of the DMN was reduced or altered in patients with AD (Kvavilashvili et al., 2020; Mahady et al., 2021; Wang et al., 2021). Resting-state fMRI of patients with amnestic mild cognitive impairment (aMCI) revealed that, compared with normal subjects, aMCI patients showed decreased activity in the lateral prefrontal cortex, left medial temporal lobe, PCC/PCUN, and right AG of the DMN, and the activity in the left lateral prefrontal cortex, left PCUN, and right AG of the DMN was positively correlated with memory scores. These findings suggest that changes in memory in patients with aMCI are associated with changes in DMN activity (Jin et al., 2012). Studies have shown that FC in the DMN is associated with episodic memory and processing speed but not with working memory or executive function. FC changes in the DMN can be used as a predictor of episodic memory performance during aging (Staffaroni et al., 2018). Resting-state fMRI of normal older adults showed that the immediate recall of the AVLT was positively correlated with regional homogeneity (ReHo) in the PCC/PCUN of normal older adults and was significantly associated with the FC of the PCC/PCUN in the DMN (Huo et al., 2018). A PET-based study on cerebral glucose metabolism and memory decline in older adults also showed significant correlations of glucose metabolism in the bilateral PCC and left PCUN with delayed recall scores (Brugnolo et al., 2014). In light of the above observations, our findings revealed that the FC of the bilateral PCUN in the DMN was significantly elevated after rTMS intervention. Similar to previous studies, this result indicated that rTMS acting on the DLPFC can regulate FC within DMN (Xue et al., 2017; Shang et al., 2018, 2020). Since the target site, the DLPFC, isalso part of DMN (Raichle et al., 2001; Greicius et al., 2003). Similarly, we also found that the DMN network of the elderly included part of bilateral DLPFC in this study. In consequence, the within-DMN FC changes suggest that the HF-rTMS effects are constrained by the brain network in which the target site locates (Shang et al., 2020). Regardless of the apparent network-wise constraint, another reason for HF-rTMS only affecting DMN might be related to the role of DMN, which is the most prominent network presenting coherent activity during the resting-state. Furthermore, the FC changes of the left PCUN were positively correlated with the AVLT score of immediate recall. These results suggest that HF-rTMS may improve immediate recall memory in older adults by regulating the FC changes in the DMN. Such intervention may be achieved by modulating the metabolism in the PCUN, which is a core region in the DMN (Utevsky et al., 2014) and plays an important role in memory retrieval, self-awareness, and visuospatial function (Cavanna and Trimble, 2006; Zhang and Li, 2012). Structure and task-based fMRI studies have linked the PCUN to memory and visuospatial impairment in AD (Karas et al., 2007; Sperling et al., 2010). Furthermore, our results related to the previous study indicate that rTMS improves verbal memory in AD patients by acting on the PCUN (Koch et al., 2018). The improvement of verbal memory in AD patients may be related to the regulation of FC between the PCUN and DMN by rTMS.

Inconsistent with previous research, there were no significant interaction effects observed in the FPCN (Li et al., 2017; Xue et al., 2017; Schluter et al., 2018; Yuan et al., 2021). There are three possible reasons for this inconsistency. First, the sample size of this study is small, which led to poor statistical effectiveness and the results could not be corrected by FDR. Secondly, the healthy subjects included in this study were 65–75 years old, the pattern of connectivity within the FPCN was different in healthy older adults compared with younger adults or patients with MCI. More importantly, most of the above studies only selected the left DLPFC for stimulation, Schluter’s study found that stimulation of the left DLPFC resulted in decreased FC in the SN whereas right stimulation resulted in an increased FC within this network, which suggests that left and right DLPFC HF-rTMS may have differential effects on resting-state functional brain network. And consistent with our findings, Schluter also found no differences between left or right DLPFC stimulation in between network connectivity (Schluter et al., 2018). The differences between bilateral DLPFC and unilateral stimulation need more research in the future.

This study has some shortcomings. The sample size of this study is small. Therefore, we are cautious about the results that HF-rTMS over the DLPFC cannot change the FC within or between brain functional networks. And the regulation of rTMS on brain activity may also be related to structural connectivity, which is our future research. Secondly, this study was conducted in older adults, and we are not sure whether these brain network differences and correlations will exist in the younger population. Specifically, the study of rTMS and its effects on brain networks and verbal memory in older adults was suspended due to the outbreak of the COVID-19 pandemic. The changes in brain networks and verbal memory in healthy older adults at 2 and 6 months after HF-rTMS will be investigated after the remission of the pandemic to further explore the mechanism of action of rTMS on cognitive aging in the long term.

## Conclusions

The results of this study suggest that HF-rTMS can improve verbal memory function in older adults and modulate FC in the DMN. Furthermore, the improvement of the immediate recall function may be related to the FC changes of the left PCUN within the DMN. These findings suggest that HF-rTMS treatment is useful and safe in the early stage of cognitive aging, which provides an effective treatment option for aging-related cognitive dysfunction.

## Data Availability Statement

The original contributions presented in the study are included in the article/\hyperref[s10]**Supplementary Material**, further inquiries can be directed to the corresponding author/s.

## Ethics Statement

The studies involving human participants were reviewed and approved by The local ethics committees of Institute of Biomedical Engineering, Chinese Academy of Medical Sciences and Peking Union Medical College. The patients/participants provided their written informed consent to participate in this study.

## Author Contributions

DC wrote the first draft of the manuscript. JJ, XW, and YL conducted the statistical analyses. DC, WC, and HW conducted the MRI analyses. ZL, TL, and TY designed the study and had full access to all the data in the study and took responsibility for the integrity of the data and the accuracy of the data analysis. All authors contributed to the article and approved the submitted version.

## Conflict of Interest

The authors declare that the research was conducted in the absence of any commercial or financial relationships that could be construed as a potential conflict of interest.

## Publisher’s Note

All claims expressed in this article are solely those of the authors and do not necessarily represent those of their affiliated organizations, or those of the publisher, the editors and the reviewers. Any product that may be evaluated in this article, or claim that may be made by its manufacturer, is not guaranteed or endorsed by the publisher.

## Footnotes

^1^ http://www.fil.ion.ucl.ac.uk/spm/

^2^ http://rfmri.org/DPARSF

^3^ https://trendscenter.org/software/
