# Supplementary material for: Beneficial Effect of High-Frequency Repetitive Transcranial Magnetic Stimulation for the Verbal Memory and Default Mode Network in Healthy Older Adults
Source: Front Aging Neurosci. 2022 May 4;14:845912. doi: 10.3389/fnagi.2022.845912 (PMC9114775; doi:10.3389/fnagi.2022.845912)
Supplement: Supplementary file 1 [file Data_Sheet_1.docx]

Supplementary materials

Supplementary Table 1 Group differences in AVLT between the four groups

| Characteristics | G1 VS. G2 ^&^ | G3 VS. G4 ^&^ | G1 VS. G3 ^#^ | G2 VS. G4 ^#^ |
| --- | --- | --- | --- | --- |
| AVLT-1 | -7.102/<0.001^***^ | -1.094/0.288 | 0.242/0.626 | 6.141/0.016^*^ |
| AVLT-2 | -3.133/0.005^**^ | -0.590/0.562 | 0.111/0.741 | 3.924/0.056 |
| AVLT-3 | -5.508/<0.001^***^ | -0.584/0.566 | 0.132/0.719 | 10.02/0.003^**^ |

Note: ^*^ p < 0.05, ^**^ p < 0.01, ^***^ p < 0.001.

&, Paired T test (T value/p value); #, General linear model (GLM), age, educational level, and sex were included as covariates (F value/p value).

Abbreviations: G1, rTMS baseline; G2, rTMS intervention; G3, Sham baseline group; G4, Sham intervention.

AVLT, auditory verbal learning test. AVLT-1, Immediate recall; AVLT-2, Delayed recall (5 minutes); AVLT-3, Delayed recall (20 minutes).


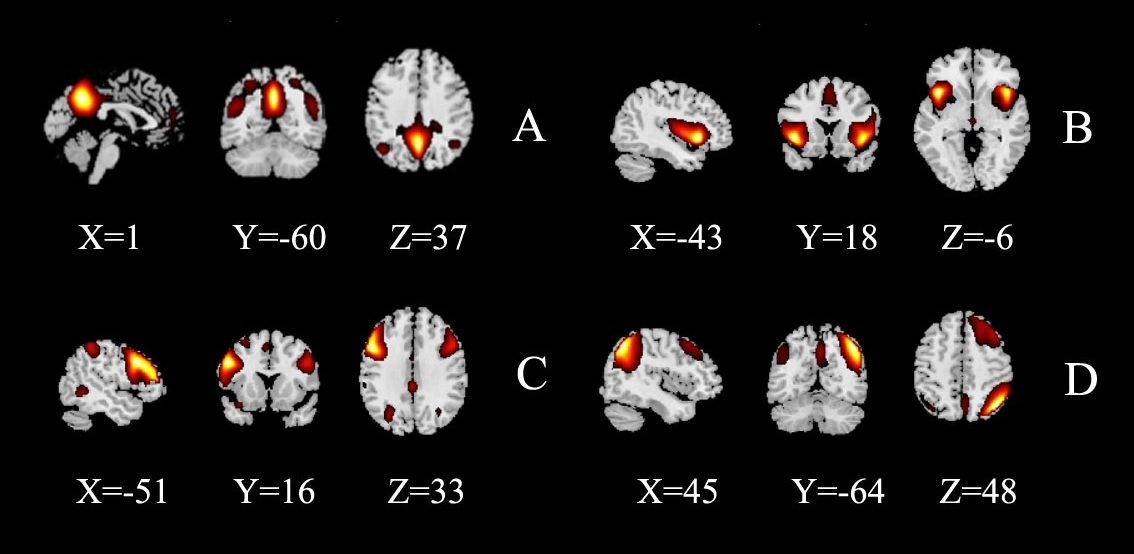


Supplementary Figure 1 Functional brain network based on ICA

A, default mode network; B, salience network; C, left frontoparietal executive control network; D, right frontoparietal executive control network.

Note: The coordinates in the figure 1 is the MNI coordinates of peak value in the functional brain network


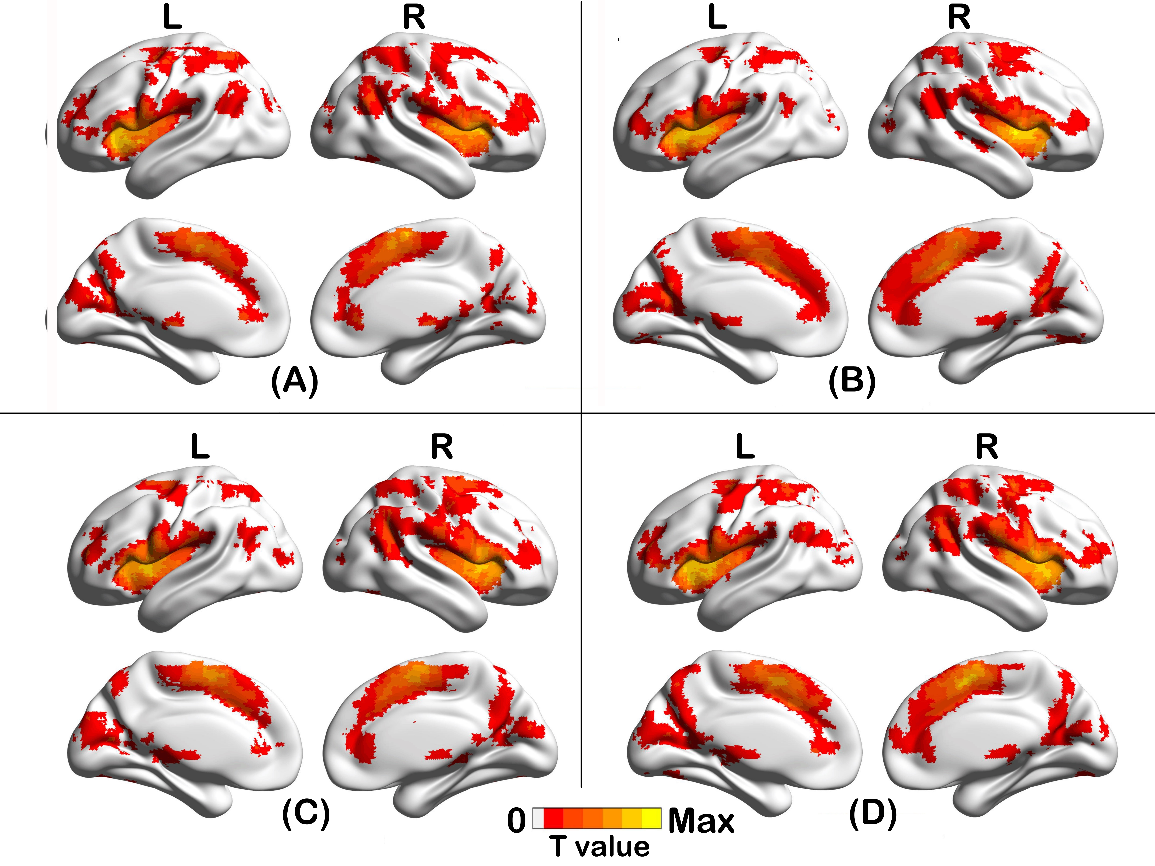


Supplementary Figure 2 One-sample T test results of the salience network. (A) rTMS baseline group, (B) rTMS intervention group, (C) Sham baseline group, (D) Sham intervention group, (p < 0.05, FDR correction, voxels > 486mm3).


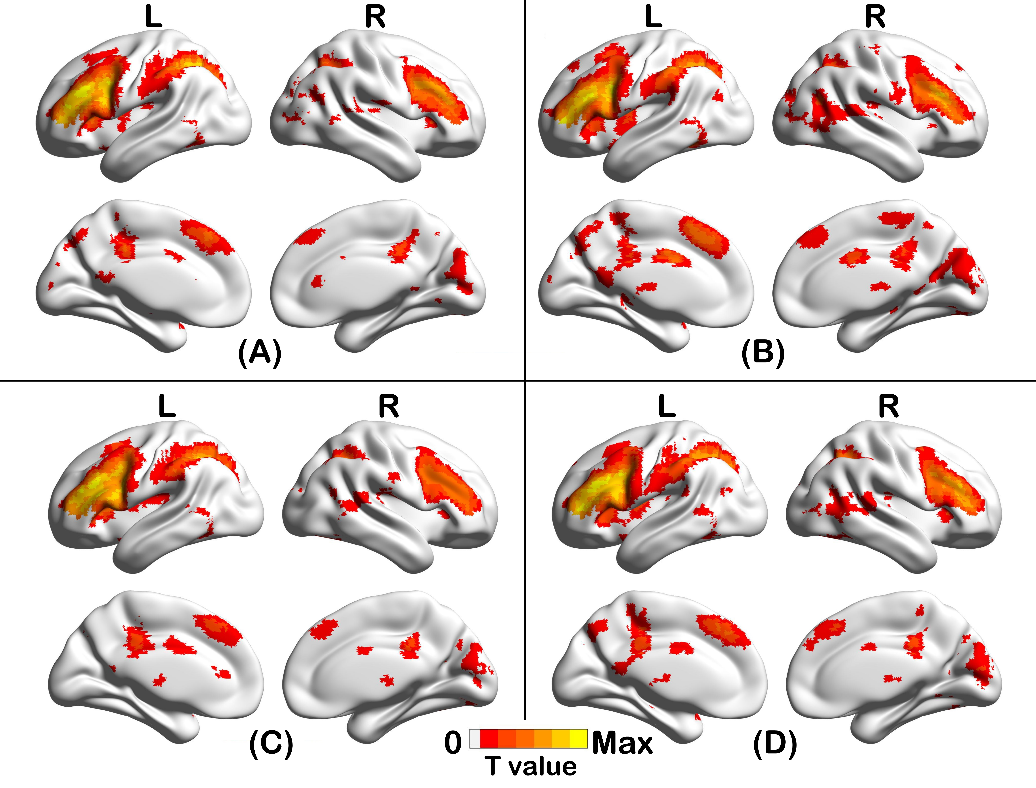


Supplementary Figure 3 One-sample T test results of the left frontoparietal executive control network. (A) rTMS baseline group, (B) rTMS intervention group, (C) Sham baseline group, (D) Sham intervention group, (p < 0.05, FDR correction, voxels > 486mm^3^).


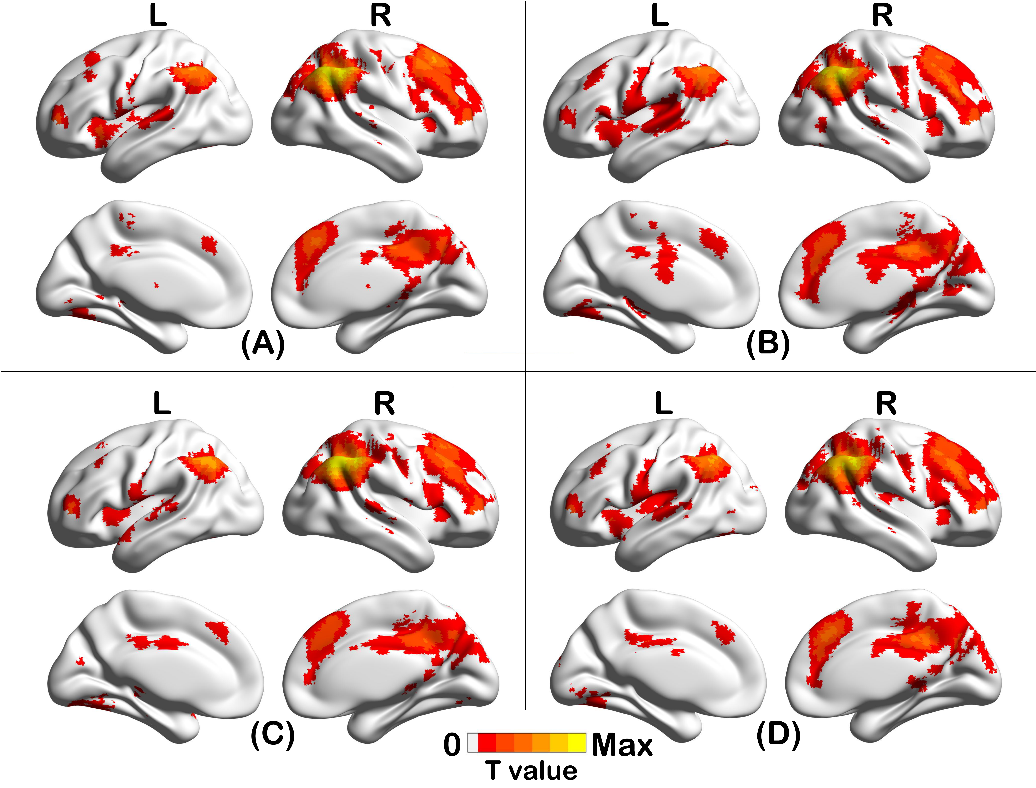


Supplementary Figure 4 One-sample T test results of the right frontoparietal executive control network. (A) rTMS baseline group, (B) rTMS intervention group, (C) Sham baseline group, (D) Sham intervention group, (p < 0.05, FDR correction, voxels > 486mm^3^).
